# Renal cancer and pneumothorax risk in Birt–Hogg–Dubé syndrome; an analysis of 115 *FLCN* mutation carriers from 35 BHD families

**DOI:** 10.1038/bjc.2011.463

**Published:** 2011-12-06

**Authors:** A C Houweling, L M Gijezen, M A Jonker, M B A van Doorn, R A Oldenburg, K Y van Spaendonck-Zwarts, E M Leter, T A van Os, N C T van Grieken, E H Jaspars, M M de Jong, E M H F Bongers, P C Johannesma, P E Postmus, R J A van Moorselaar, J-Htm van Waesberghe, T M Starink, M A M van Steensel, J J P Gille, F H Menko

**Affiliations:** 1 Department of Clinical Genetics, VU University Medical Center, PO Box 7057, Amsterdam 1007 MB, The Netherlands; 2 Department of Dermatology, GROW School for Oncology and Developmental Biology, Maastricht University Medical Center, Maastricht, The Netherlands; 3 Department of Mathematics, VU University, Amsterdam, The Netherlands; 4 Department of Dermatology, VU University Medical Center, The Netherlands; 5 Department of Clinical Genetics, Erasmus University Medical Center, Rotterdam, The Netherlands; 6 Department of Genetics, University Medical Center Groningen, University of Groningen, Groningen, The Netherlands; 7 Department of Clinical Genetics, Academic Medical Center, Amsterdam, The Netherlands; 8 Department of Pathology, VU University Medical Center, The Netherlands; 9 Department of Human Genetics, Radboud University Nijmegen Medical Center, Nijmegen, The Netherlands; 10 Department of Pulmonology, VU University Medical Center, the Netherlands; 11 Department of Urology, VU University Medical Center, The Netherlands; 12 Department of Radiology, VU University Medical Center, The Netherlands

**Keywords:** Birt–Hogg–Dubé syndrome, folliculin, fibrofolliculoma, renal cancer, discoid fibroma, pneumothorax

## Abstract

**Background:**

Birt–Hogg–Dubé (BHD) syndrome is an autosomal dominant condition caused by germline *FLCN* mutations, and characterised by fibrofolliculomas, pneumothorax and renal cancer. The renal cancer risk, cancer phenotype and pneumothorax risk of BHD have not yet been fully clarified. The main focus of this study was to assess the risk of renal cancer, the histological subtypes of renal tumours and the pneumothorax risk in BHD.

**Methods:**

In this study we present the clinical data of 115 *FLCN* mutation carriers from 35 BHD families.

**Results:**

Among 14 *FLCN* mutation carriers who developed renal cancer 7 were <50 years at onset and/or had multifocal/bilateral tumours. Five symptomatic patients developed metastatic disease. Two early-stage cases were diagnosed by surveillance. The majority of tumours showed characteristics of both eosinophilic variants of clear cell and chromophobe carcinoma. The estimated penetrance for renal cancer and pneumothorax was 16% (95% minimal confidence interval: 6–26%) and 29% (95% minimal confidence interval: 9–49%) at 70 years of age, respectively. The most frequent diagnosis in families without identified *FLCN* mutations was familial multiple discoid fibromas.

**Conclusion:**

We confirmed a high yield of *FLCN* mutations in clinically defined BHD families, we found a substantially increased lifetime risk of renal cancer of 16% for *FLCN* mutation carriers. The tumours were metastatic in 5 out of 14 patients and tumour histology was not specific for BHD. We found a pneumothorax risk of 29%. We discuss the implications of our findings for diagnosis and management of BHD.

Birt–Hogg–Dubé syndrome (BHD, OMIM #135150) is an autosomal dominant condition characterised by fibrofolliculomas, pneumothorax and renal tumours. BHD is caused by germline mutations in the *FLCN* gene encoding folliculin (Nickerson *et al*, 2002). In the original kindred skin lesions were the only clinical manifestation (Birt *et al*, 1977). Subsequently, renal cancer and pneumothorax were found to be part of the syndrome (Roth *et al*, 1993; Toro *et al*, 1999). Furthermore, the risk for colorectal cancer might be slightly increased in *FLCN* mutation carriers (Nahorski *et al*, 2010). The functions of folliculin have partly been clarified and might include a role in the mammalian target of rapamycin pathway (Hasumi *et al*, 2009). Although many BHD kindreds exhibit all three components of the syndrome, ‘pneumothorax-only’ and ‘renal-cancer-only’ families have also been described (Graham *et al*, 2005; Painter *et al*, 2005; Woodward *et al*, 2008; Kunogi *et al*, 2010). Among 69 patients with early-onset or familial clear cell renal cancer without further characteristics of BHD, germline *FLCN* mutations were found in 4% of cases (Woodward *et al*, 2008). In recent reviews, the variable clinical manifestations, molecular pathogenesis and management options for BHD were summarised (Schmidt *et al*, 2005; Toro *et al*, 2008; Menko *et al*, 2009).

For optimal early detection and treatment of BHD-associated renal cancer, insight into the renal cancer risk and the clinical picture of these tumours is essential. Among cohorts of BHD patients a wide range of prevalence of kidney tumours has been observed, ranging from 6.5 to 34% (Toro *et al*, 2008). The differences in prevalence are probably due to ascertainment in dermatological *vs* urological clinics and age at examination. Notably, the lifetime renal cancer risk for *FLCN* mutation carriers has not yet been established. In BHD, renal cancer is generally diagnosed at a relatively young age and commonly presents as bilateral and/or multifocal disease. The renal neoplasms typically found in BHD patients were described as hybrid tumours, containing elements of different histological subtypes, in particular chromophobe tumours and oncocytoma. However, other subtypes including clear cell renal carcinoma have also been reported (Pavlovich *et al*, 2002, 2005; Schmidt *et al*, 2005; Woodward *et al*, 2008).

A 50-fold increased risk of spontaneous pneumothorax in BHD was reported (Zbar *et al*, 2002). Among cohorts of BHD patients the prevalence of pneumothorax ranged from 24 to 38% (Schmidt *et al*, 2005; Toro *et al*, 2007, 2008). Again, ascertainment has varied for cohorts of patients and the lifetime risk of pneumothorax for *FLCN* mutation carriers has not yet been established. On CT examination of the thorax, more than 80% of adult BHD patients had multiple lung cysts, most often in the basal lung regions. The presence of lung cysts is probably related to the increased risk for pneumothorax, which is often recurrent in BHD patients (Toro *et al*, 2007). A positive family history for pneumothorax was associated with an increased risk of pneumothorax and patients with a family history positive for renal cancer had an increased risk of having renal tumours. However, a family history of renal cancer was not associated with an increased pneumothorax risk (Toro *et al*, 2008).

Previously, we described 25 *FLCN* germline mutation carriers from 11 BHD families (Leter *et al*, 2008). Here, we present an update of this cohort and add the evaluation of 24 new kindreds with pathogenic *FLCN* mutations. In total, the clinical histories of 115 *FLCN* mutation carriers from 35 BHD families have been assessed. The main focus of this study was to assess the risk of renal cancer, the histological subtypes of renal tumours and the pneumothorax risk in BHD. Furthermore, we consider the yield of *FLCN* mutation analysis and the clinical phenotype in kindreds without *FLCN* mutations.

## Patients and methods

### Ascertainment of pedigrees

The BHD database at VU University Medical Center currently lists more than 65 Dutch families referred for suspected BHD; 53 of these kindreds with completed family studies are considered in this report. In all, 40 of these 53 families were referred to our center, whereas other Dutch clinical genetics centers contributed an additional 13 families with pathogenic *FLCN* mutations (Table 1).

The index patient of 48 out of the 53 families was referred by a dermatologist after fibrofolliculomas were diagnosed. In three BHD families the index patient had renal cancer (BHD families 33, 35 and 63), in one kindred the proband had recurrent pneumothorax (BHD 29). One patient without an identifiable *FLCN* mutation was referred for multiple pulmonary cysts.

For ascertainment of pedigrees, the proband was requested to inform family members by means of a written summary letter about BHD. After completion of the initial evaluation reminders were sent to probands aimed at complete ascertainment of family members.

### Mutation analysis

After informed consent genomic DNA was extracted from blood samples. Primers for the amplification and sequencing of the 14 exons were detailed previously by Nickerson *et al* (2002). PCR amplification was performed using a PE 9700 thermocycler (Applied Biosystems, Forster City, CA, USA). Sequencing reactions were performed using Big Dye Terminator (Applied Biosystems) and run on an ABI 3100 genetic analyzer (Applied Biosystems). For the detection of deletions and duplications of one or more exons the SALSA MLPA kit P256 obtained from MRC Holland was used (http://www.mrc-holland.com).

### Statistical analysis

Conditional on the mutation status for different individuals, we assumed the various expressions of the BHD phenotype to be mutually independent and that for individual cases the risks for renal cancer and pneumothorax and the ages at which these traits were expressed are independent. Finally, the penetrances of renal cancer and pneumothorax were assumed to be equal for male and female mutation carriers.

For the estimates of the penetrance of pneumothorax and renal cancer we included 21 out of 22 *FLCN*-positive pedigrees investigated at our center. For these families medical records of all mutation carriers and information on untested relatives were available. Out of these 21 pedigrees, 20 were ascertained via a proband referred by a dermatologist. One proband (BHD 29) was referred by a pulmonologist for analysis of recurrent pneumothorax. Therefore, we included the proband data in the estimation of renal cancer and pneumothorax risks, with the exception of the proband referred for analysis of pneumothorax.

**Figure 1:**
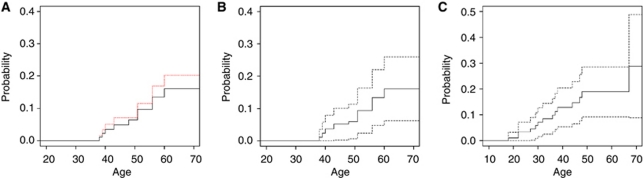
(**A**) Estimate of the age-related penetrance function for renal cancer based on available data on both mutation carriers and their untested relatives till the age of 70: 16% (continuous line *n*=86 mutation carriers and 84 untested relatives), together with the Kaplan–Meier estimator based on the known mutation carriers only at age 70: 20% (*n*=86, red dashed line). (**B**) Estimate of the age-related penetrance function for renal cancer based on available data on both mutation carriers and their untested relatives together with a minimum 95% confidence interval. Estimated penetrance at age 70: 16%, 95% minimal confidence interval: (6-26%). (**C**) Estimation of the penetrance function of age at the first pneumothorax and a minimum 95% confidence interval based on available data on both mutation carriers and their untested relatives (*n*=85 mutation carriers and 84 untested relatives). Estimated penetrance at age 70: 29%, 95% minimal confidence interval: (9–49%).

Major problems of penetrance estimates are missing data and possible preferential testing of individuals affected with complications of BHD, that is, renal cancer or pneumothorax. Indeed, if the mutation status and phenotype had been known for all individuals in all pedigrees the penetrance curves could have been assessed using the Kaplan–Meier estimator. Unfortunately, however, the mutation status or phenotype was not known for all family members. Excluding the individuals for whom the mutation status was unknown would likely lead to an overestimation of risk (see Figure 1A), assuming that non-affected individuals may be less willing to be genotyped. For our corrected risk estimation we assumed that a negative family history for renal cancer or pneumothorax in a close relative indeed reflects the absence of these complications in the untested relative. We imputed the missing data as follows: for every individual in the data set who was not genotyped the probability that he or she was a carrier was computed on the basis of the mutation status of his/her relatives. Subsequently, using this carrier probability it was sampled whether he or she was a mutation carrier or not. As a result, for every individual the mutation status was assigned and in combination with the phenotype Kaplan–Meier curves and confidence intervals were computed. This strategy of sampling the mutation status and estimating the penetrance functions by the Kaplan–Meier estimator was repeated 10 000 times. Next, the mean of the 10 000 estimated curves and upper and lower bounds of the confidence intervals were computed. These curves are plotted in Figure 1A and B for renal cancer and in Figure 1C for pneumothorax. By using this strategy the estimator will be asymptotically unbiased. The confidence interval found is slightly too narrow because there is a greater degree of uncertainty than would have been the case if the missing mutation statuses had actually been observed (as assumed after imputation). Therefore, the final confidence interval has a confidence of slightly less than 95%. Although the exact confidences of the intervals are not exactly known we included the calculated intervals in the figures in order to make those who would use the figures for consultation aware of the uncertainty of the given risk estimates.

## Results

**Figure 2:**
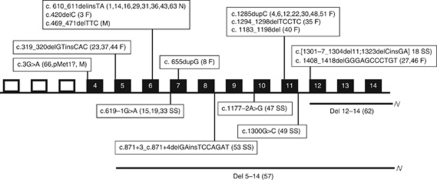
Overview of the *FLCN* mutations identified in this study. Del: deletion, F: frameshift, M: missense, N: nonsense, SS: splice site. Exons are depicted as rectangles. The family numbers are shown after the mutations.

The characteristics of 53 families referred for suspected BHD are presented in Table 1. The main features of the BHD kindreds with pathogenic *FLCN g*ermline mutations are listed in Table 2. The mutations detected are depicted in Figure 2. Data on the renal tumours and pneumothorax are given in Tables 3 and 4, respectively. Among the 27 families with clinical BHD based on dermatological evaluation at our center, 22 had pathogenic *FLCN* mutations (mutation detection rate 81%). Characteristics of the five families without a detectable *FLCN* mutation are summarised in Table 5; they include two families with an unclassified exon 1 deletion. Analysis of these exon 1 deletions is ongoing. In the cohort of our current study, no colorectal cancer was reported. Colorectal adenomas were diagnosed by colonoscopy in four *FLCN* mutation carriers (age 40, 42, 83, these patients were also included in our previous reports by Leter *et al*, 2008 and Nahorski *et al*, 2010 and age 44, not reported previously).

Other malignancies than renal cancer were reported in six patients previously documented in Leter *et al* (2008). In addition to the patients reported by Leter *et al* (2008) in five patients without renal cancer, tumours were reported including non-melanoma skin cancer in two patients and single cases of melanoma, sarcoma, bladder and prostate cancer. The patient diagnosed with melanoma was also diagnosed with sarcoma. Three patients with renal cancer were diagnosed with additional tumours. One patient had oncocytic pituitary adenoma at age 9, astrocytoma at age 12, pheochromocytoma at age 34 and renal cancer at age 34 and 35 (BHD 63, Table 3). No evidence for other tumour susceptibility was found. The other two patients had gastric carcinoma at age 55 and renal cancer at age 56 (BHD 43, M, Table 3) and prostate carcinoma and renal cancer at age 50 (BHD 46, Table 3), respectively.

### Renal manifestations

**Figure 3:**
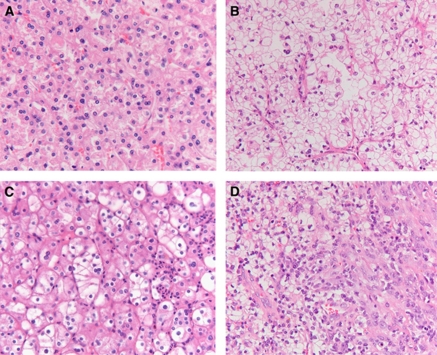
Illustration of the histological pictures of renal cell carcinomas in our series. (**A**) The most common pattern found, classified as clear cell/chromophobe. The tumour cells show eosinophilic cytoplasm, moderate nuclear pleiomorphism, vague perinuclear halos, no explicit cell borders and no vascular prominence. (**B**) Classical picture of clear cell carcinoma, as can be seen in many of the tumours in our series, but mostly only in part of the tumour cells. (**C**) A clear cell/chromophobe renal cell carcinoma with partially clear cytoplasm, more prominent cell borders, no thick-walled vessels. (**D**) Renal cell carcinoma with sarcomatoid changes (in the right part of the picture).

In all, 14 out of 115 (12%) *FLCN* mutation carriers from 12 families were diagnosed with renal cancer. All available tumours were revised at our center by two pathologists. In addition, one mutation carrier had renal oncocytoma (BHD 57). Five mutation carriers died of metastatic renal cancer (BHD 6, BHD 16, BHD 23, BHD 37 and BHD 44; Table 3). All were diagnosed with renal cancer after symptoms had developed. The histological classification of the renal tumours according to the WHO criteria is shown in Table 3 (Lopez-Beltran *et al*, 2009). Most of the tumours showed cells with granular/floccular eosinophilic cytoplasm, as can be seen in both clear cell carcinoma (formerly called the eosinophilic variant, Table 3) and chromophobe carcinoma. This eosinophilic cell variation is also often seen in sporadic clear cell carcinoma (Kummerlin *et al*, 2009; Lopez-Beltran *et al*, 2009). As most tumours had mainly eosinophilic cytoplasm, with moderately sharp cell borders, a vague perinuclear halo and moderately enlarged nuclei, we classified them as intermediate between clear cell and chromophobe carcinoma (CC/Cph) (Table 3 and Figure 3). One of the tumours showed sarcomatoid changes, which can develop in both clear cell and chromophobe carcinoma (Table 3, BHD 16). One other tumour had papillary structures in combination with clear cell changes (Table 3, BHD 6). Two additional patients from *FLCN* positive families but with unknown mutation carrier status (BHD 1 and BHD 42) were diagnosed with renal cancer. These two tumours also showed mixed chromophobe/clear cell histology. In all, 2 of the 14 renal tumours (BHD 1 and BHD 32, Table 3) were detected by surveillance. For one of these patients renal cancer was detected on the first ultrasound performed after the diagnosis BHD was made (BHD 32, Table 3). For the other patient renal cancer was detected by ultrasound 4 years after the preceding normal ultrasound/MRI (BHD 1, Table 3); the latter patient did not undergo standard yearly surveillance after the initial imaging.

### Cutaneous manifestations

Most *FLCN* mutation carriers (91/115, 79%), underwent dermatological evaluation (68 of which were evaluated by dermatologists at our center). In all 19 (21%) cases, aged 23–72 years, had no cutaneous abnormalities; 14 (74%) of the patients without cutaneous manifestations were over age 40. Notably, one patient who presented with metastatic renal cancer at age 51 (BHD 23) had no cutaneous lesions. The youngest mutation carrier with histologically confirmed fibrofolliculomas was 25 years old (BHD 23). In five families without an identifiable *FLCN* mutation (BHD 2, 5, 9, 25 and 28) the clinical diagnosis BHD was based on histologically confirmed multiple fibrofolliculomas; in one of these kindreds both pneumothorax and renal cancer occurred (BHD 25, Table 5). In seven families without *FLCN* mutations the skin lesions consisted of multiple discoid fibromas of childhood onset (families 11, 17, 20, 24, 38, 45 and 50). In two of these families (24 and 45) a genealogical study showed common ancestry. No renal or pulmonary signs were present in these seven families, except for one case with pneumothorax. For two of these families the *FLCN*-locus was excluded by linkage analysis (Starink *et al*, 2011).

### Pulmonary manifestations

Among the 115 *FLCN* mutation carriers, 28 (24%) had a history of pneumothorax, recurrent in eight patients (Table 4). In all, 4 out of 28 patients with a previous pneumothorax were confirmed (former) smokers. The medical records of the other patients did not state a history of smoking. The mean age of the first pneumothorax was 36 years (range 18–74 years). We did not systematically subject mutation carriers to CT scanning of the lungs. For 12 *FLCN* mutation carriers the report of a CT-scan of the thorax was available. Scans were performed either to confirm suspected BHD or because of pulmonary complications of BHD. In five of these patients (aged 25–46 years), multiple cysts were reported in one or both lungs. Of these, two had a history of recurrent or bilateral pneumothorax before the age of 30 years. In seven *FLCN* mutation carriers no pulmonary cysts were detected (age 23–71, four were over age 40).

### Cumulative renal cancer and pneumothorax risk

The estimated renal cancer penetrance based on assessment of *FLCN* mutation carriers only was 20% at age 70 (red dashed line, Figure 1A). In contrast, when considering both tested and untested relatives, the estimated renal cancer penetrance at age 70 was 16% (continuous line, Figure 1A and B). The estimated penetrance for the first episode of spontaneous pneumothorax was 29% (95% minimal confidence interval: 9–49%, Figure 1C) at 70 years, again considering information on both mutation carriers and their untested relatives.

## Discussion

An important aim of this study was the estimation of renal cancer and pneumothorax penetrance in BHD. By incorporating data on relatives who did not undergo DNA testing we found an estimated penetrance for renal cancer of 16% and a penetrance for pneumothorax of 29% at the age of 70 years. The wide range of prevalence for renal cancer among cohorts of BHD reported in literature is 6.5–34% (Toro *et al*, 2007, 2008; Kunogi *et al*, 2010). The renal cancer risk we found of around 16% at age 70 years is important for counselling of *FLCN* mutation carriers and their families ascertained in cancer family clinics. Future studies with larger patient groups and comparison between cohorts investigated in different populations may lead to further specification of the renal cancer risk.

The clinical presentation, histological pattern and biological behaviour of renal cancer in *FLCN* mutation carriers are important for several reasons. First, a pathognomonic histological pattern would be helpful for early diagnosis. Previously, The European BHD Consortium proposed clinical diagnostic criteria for BHD, which included early-onset, bilateral and multifocal renal tumours and a mixed chromophobe and oncocytic histological pattern. In addition, *FLCN* mutation analysis should be considered for patients who have familial cystic lung disease, familial pneumothorax, familial renal cancer, or any combination of spontaneous pneumothorax and kidney cancer (Menko *et al*, 2009).

Notably, among 14 BHD patients in the current study who developed renal cancer, clinical signs of hereditary disease (age at onset <50 years and/or multifocal/bilateral tumours) were present in only seven cases. In addition, the histological picture of BHD-associated renal cancer in this cohort was not typical for this syndrome. In all, 10 out of 14 renal tumours revised in this series were difficult to classify. None of them showed classical features of chromophobe renal cell carcinoma. Instead, they mainly exhibited characteristics of both eosinophilic variants of clear cell cancer and chromophobe carcinoma. One of the tumours was a hybrid form of clear cell and papillary carcinoma and one showed sarcomatoid changes. These histological patterns can also be found in sporadic RCC. Therefore, late-onset unilateral, unifocal clear cell renal cancer does not exclude BHD.

Furthermore, although BHD-associated renal tumours were reported to metastasise rarely (Toro *et al*, 2008), 5 of the 14 patients with renal cancer in our cohort developed metastatic disease underlining the importance of early detection of these renal tumours by surveillance. In recently diagnosed families not included in this report, we observed two BHD patients with renal cancer at the ages of 30 and in the early twenties, respectively, underlining that surveillance for renal cancer should be offered to BHD patients from early age onward.

Of the 115 *FLCN* mutation carriers 28 had a history of pneumothorax, frequently recurrent and bilateral. The mean ages at which pneumothorax and renal cancer occurred in our cohort were 36 and 49 years, respectively. Among the 14 renal cancer patients described five had a history of pneumothorax (Tables 3 and (4), preceding renal cancer by several years in three patients (BHD 32, 37, 43, Table 4). Although an increased risk for renal cancer in BHD families with a positive history for pneumothorax has not been observed (Toro *et al*, 2008), bilateral, recurrent or familial pneumothorax may serve as an early indicator of BHD syndrome (Johannesma *et al*, 2009).

Ever since Hornstein and Knickenberg (1975) described the combination of skin fibrofolliculomas and colorectal polyps it is a matter of debate whether BHD is associated with an increased risk of colorectal neoplasia. Although Zbar *et al* (2002) found no significantly increased risk Khoo *et al* (2002) proposed that the risk might apply to specific subgroups only. Nahorski *et al* (2010) found evidence that the risk may be dependent on the *FLCN* genotype. Recently, in family BHD15, one of the *FLCN* mutation carriers developed symptomatic colonic cancer at age 62 years. Late-onset colorectal cancer has been diagnosed in several other *FLCN* mutation carriers from our BHD cohort not included in the present study. Although colorectal cancer may well be coincidental in these cases the current data call for further evaluation of the colorectal neoplasia risk in BHD.

Among 27 families with clinical BHD (multiple fibrofolliculomas) evaluated at our center 22 had pathogenic *FLCN* mutations, resulting in a 81% yield for *FLCN* mutation analysis. Four patients referred for suspected BHD declined genetic testing.

In two of the families (BHD 2 and 25) with clinical BHD but without a mutation in the coding region of *FLCN*, a deletion of exon 1 was observed. Exon 1 is the first of three non-coding exons. The exact size and the effect of these deletions remain to be determined. Recently, after completion of our study, intragenic deletions and a duplication were reported in patients with BHD (Benhammou *et al*, 2011). Using a luciferase reporter assay, this study also showed that the expression was strongly reduced when exon 1 was deleted. Analysis of the pathogenicity and co-segregation of the deletions detected in our families are currently ongoing to prove pathogenicity. Therefore, these families were not included in the calculation of the penetrance for renal cancer and pneumothorax, although the deletions are very likely to be pathogenic. In the current study, intragenic deletions were detected in two additional BHD families (BHD 57, 62), underlining the importance of MLPA or CGH analysis in patients with a clinical suspicion of BHD without an identifiable *FLCN* mutation.

Nine patients referred for possible BHD were diagnosed with other conditions. One patient had probable tuberous sclerosis and one patient had pulmonary emphysema. Seven of the families were diagnosed with familial multiple discoid fibromas (FMDF). In 1985, one of the authors described this entity as a dominant condition distinct from BHD, showing childhood onset, preferential localisation of lesions on the ears and distinct histology, which mimics the trichodiscomas in BHD (Starink *et al*, 1985). Thus far no systemic complications have been noted for FMDF except for one patient with pneumothorax. We have now excluded involvement of the *FLCN* locus in two FMDF kindreds using linkage analysis (Starink *et al*, 2011).

Currently, we use the renal cancer risk of around 16% by age 70 years for the counselling of patients to emphasise the importance of surveillance for renal cancer. As according to our histological data, the renal cancers found in BHD were not evidently different from sporadic tumours, future studies aimed at the classification of BHD-associated renal cancer in comparison with sporadic disease are essential. Both the renal cancer risk and the pneumothorax risk (about 16% and 29% at age 70 years, respectively) are based on a large set of data using a model incorporating available data of family members not subjected to DNA testing. Evaluation of larger patient groups and patients from other populations may yield other penetrance figures in the future.

The European BHD Consortium (Menko *et al*, 2009) proposed *FLCN* mutation testing in patients with early-onset renal cancer (<50 years), in particular with multifocal or bilateral disease (or both) with chromophobe or oncocytic histology and in familial renal cancer cases. Age at diagnosis of (the first) renal cancer in our patient group was at or above 50 years of age in half of the patients and the tumour histology was mixed in most patients but included clear cell elements in all cases. Therefore, it will be important to study the yield of *FLCN* mutation testing using a wider set of criteria than proposed previously. The histology and molecular pathology of renal tumours are associated with their biological behaviour and reaction to systemic treatment of metastatic disease. Therefore, additional studies are needed to monitor the success of surveillance for renal cancer, the results of surgical or other forms of local treatment such as radiofrequency ablation and response to targeted therapies.

## Figures and Tables

**Table 1:** Ascertainment of 115 *FLCN* mutation carriers from 35 BHD families considered in this study

**Subdivision of families**	**Clinical BHD and *FLCN* mutation**	**Clinical BHD without *FLCN* mutation**	**Other diagnoses**	**Evaluation declined**
40 families referred to our center for suspected BHD^a^,b	22 probands and 67 mutation-positive family members	5	9^c^	4
				
13 *FLCN*-mutation-positive families from other centres	13 probands and 13 mutation-positive family members			

a Including 12 families also described by Leter *et al*, 2008 and Johannesma *et al*, 2009.

b For calculation of renal cancer and pneumothorax penetrance the data of 86 *FLCN* mutation carriers from 21 kindreds for which complete family data were available were used.

c Seven families (11, 17, 20, 24, 38, 45 and 50) had familial multiple discoid fibromas, described by Starink *et al* (2011); two index patients (26 and 39) were diagnosed with pulmonary emphysema and probable tuberous sclerosis complex, respectively.

**Table 2:** Main features of 115 *FLCN* mutation carriers from 35 BHD kindreds with pathogenic *FLCN* germline mutations

**Family no.**	***FLCN* germline mutation**	**No. of *FLCN* mutation carriers**	**No. of mutation carriers with pneumothorax**	**No. of mutation carriers with renal cancer**
BHD 1^a^	c.610_611delinsTA	6	2	1
BHD 3^a^	c.420delC	2	0	0
BHD 4^a^	c.1285dupC	1	0	0
BHD 6^a^	c.1285dupC	4	0	2
BHD 8^a^	c.655dupG	2	0	0
BHD 12^a^	c.1285dupC	3	0	0
BHD 14^a^	c.610_611delinsTA	3	1	0
BHD 15^a^	c.619-1G>A	4	1	0
BHD 16^a^	c.610_611delinsTA	18	2	1
BHD 18^a^	c.[1301-7_1304del11; 1323delCinsGA]	2	1	0
BHD 19^a^	c.619-1G>A	1	0	0
BHD 22	c.1285dupC	1	0	0
BHD 23	c.319_320delGTinsCAC	11	1	1
BHD 27	c.1408_1418delGGGAGCCCTGT	1	0	0
BHD 29^b^	c.610_611delinsTA	2	2	0
BHD 30	c.1285dupC	1	0	0
BHD 31	c.610_611delinsTA	1	0	0
BHD 32	c.469_471delTTC	3	3	1
BHD 33	c.619-1G>A	2	1	1
BHD 35	c.1749_1753del	2	1	1
BHD 36	c.610_611delinsTA	1	0	0
BHD 37	c.319_320delGTinsCAC	15	2	1
BHD 40	c.1183_1198del	1	0	0
BHD 43	c.610_611delinsTA	5	5	2
BHD 44	c.319_320delGTinsCAC	2	0	1
BHD 46	c.1408_1418delGGGAGCCCTGT	6	0	1
BHD 47	c.1177-2A>G	2	0	0
BHD 48	c.1285dupC	1	0	0
BHD 49	c.1300G>C	1	0	0
BHD 51	c.1285dupC	1	0	0
BHD 53	c.871+3_871+4delGAinsTCCAGAT	1	0	0
BHD 57	c.250−?_1740+?del (del exon 5–14)	3	2	0
BHD 62	c.1301−?_1740+?del (del exon 12–14)	3	3	0
BHD 63	c.610_611delinsTA	2	1	1
BHD 66	c.3G>A	1	0	0

a Described by Leter *et al*, 2008.

b Described by Johannesma *et al*, 2009.

**Table 3:** Main features of 17 renal cancers in 14 *FLCN* germline mutation carriers from 12 BHD kindreds

				**Clinical characteristics^a^**					
**Family no.**	**M/F**	**Age at diagnosis (years)**	**Diagnosis^b^**	**Localisation**		**Histology**	**TNM**	**Treatment**	**Outcome^c^**
BHD 1^d^	F	60	B	1 (L)	1	CC/Cph	T1N0M0	T	A (3)
BHD 6^d^	M	39	A	1 (R)	1	Pap/CC	T3N2M1	T & Im	D (40)
	F	40	A^e^	1 (L)	2	CC/Cph	T2N0M0	T	A (7)
BHD 16^d^	M	56	A	1 (R)	1	CC/Cph/Sa	T3N0M1	T & Ch	D (57)
BHD 23	M	51	A	1 (L)	1	CC/Cph	T1N0M1	T & Ch and Rth	D (52)
BHD 32	F	51	B	1 (R)	1	CC/Cph	T1N0M0	P	A (3)
BHD 33	M	48	A	2 (L)	1	CC/Cph	T3N0M0	T	A (6)
		51	B	(R)	1	Unknown	T1N0M0	P	
BHD 35	M	38	A	2 (R)	2	CC	T1N0M0	P	A (1)
		40	B	(L)	2	Unknown	T1N0M0	P	
BHD 37	F	52	A	1 (L)	1	CC	TxNxM1	Rth	D (52)
BHD 43	F	74	A	1 (L)	2	CC/Cph	T1N0M0	T	A (3)
	M	56	A	1 (R)	2	CC/Cph	T1N0M0	T	A (2)
BHD 44	F	43	A	1 (L)	1	CC/Cph	TxNxM1	T and Me	D (57)
BHD 46	M	50	A	1 (L)	1	Unclassified^f^	T1N0M0	—	D (50)
BHD 63	M	34	A	2 (L)	2	CC/Cph	T2N0M0	T	A (3)
		35	B	(R)	1	CC/Cph	T1N0M0	P	

Abbreviations: Ad=adenocarcinoma, classification not certain; BHD=Birt–Hogg–Dubé CC=clear cell; CC/Cph=renal cell carcinoma with eosinophilic cytoplasm and characteristics of both CC and Cph; Ch=chemotherapy; Cph=chromophobe; F=female; Im=immunotherapy; P=partial nephrectomy; M=male; Pap=papillary; Rth=radiotherapy; Me=metastasectomy; Sa=sarcomatoid component; T=total nephrectomy; TNM=classification according to tumour/node/metastasis status.

a Localisation: left column: 1/2: unilateral/bilateral, in parentheses: L/R: left-sided/right-sided, right column: 1: unifocal, 2: multifocal. Histology: according to Lopez-Beltran *et al*, 2009.

b A: diagnosis after symptoms had developed; B: diagnosis after positive renal imaging of an asymptomatic individual.

c A: alive, D: deceased; in parentheses: number of years of follow-up and age at death, respectively.

d Described by Leter *et al*, 2008.

e Coincidental finding at medical examination for gastrointestinal complaints.

f The tumour was found during autopsy and could not be reliably subclassified due to autolysis.

**Table 4:** Main features of pneumothorax in 28 *FLCN* germline mutation carriers from 15 BHD kindreds

			**Clinical characteristics**		
**Family no.**	**M/F**	**Age at diagnosis**	**R/L**	**Episodes**	**Renal cancer**
BHD 1^a^	F	44	L	1	
	M	30	R	1	
BHD 14^a^	M	36	L	1	
BHD 15^a^	F	U	U	1	
BHD 16^a^	M	67	L	1	
	F	47	U	1	
BHD 18^a^	M	22	U	1	
BHD 23	M	22	L	1	
BHD 29^b^	M	25	B	5	
	M	27	B	1	
BHD 32	F	39	U	1	51
	F	U	U	1	
	M	23	U	1	
BHD 33	M	U	U	1	
BHD 35	M	38	R	2	38
BHD 37	F	48	B	1	51
	F	29	R	1	
BHD 63	M	37	B	1	
BHD 62	M	53	L	7	
	M	27	L	1	
	M	42	B	3	
BHD 43	F	38	L	1	
	M	37	R	4	
	F	74	L	1	74
	M	32	L	3	55
	F	18	R	2	
BHD 57	M	31	L	1	
	F	33	R	5	

Abbreviations: L=left lung; R=right lung; M=male; F=female; B=bilateral; U=available records did not state exact age/clinical characteristics.

Age at diagnosis: Age at diagnosis of the first episode of pneumothorax.

Renal cancer: Age at diagnosis of renal cancer in patients with a history of pneumothorax.

a Described by Leter *et al*, 2008.

b Described by Johannesma *et al*, 2009. The relative diagnosed with a clear cell renal tumour was not tested for the *FLCN* mutation.

**Table 5:** Characteristics of BHD probands without an identified *FLCN* mutation

**BHD no.**	**Sex and age of proband**	**Clinical characteristics**	**Family data**	**Remarks**
BHD 2^a^	M 46 years	Multiple fibrofolliculomas on the face, neck and trunk, starting at age 33 years	Colorectal cancer in mother	Likely pathogenic *FLCN* exon 1 deletion and two polymorphisms (IVS8+36G>A and IVS9+6C>T)^b^
BHD 5^a^	M 33 years	Multiple fibrofolliculomas on thorax since childhood		Unclassified variant in *FLCN*: IVS8+36G>A
BHD 9^a^	M 34 years	More than 100 skin lesions on the face, neck and trunk, fibrofolliculomas. ulcerative colitis, sacroiliitis		
BHD 25	F 70 years	Multiple fibrofolliculomas at age 66 years	Nephew had pneumothorax and died due to renal cancer	Likely pathogenic *FLCN* exon 1 deletion
BHD 28	M 56 years	Multiple fibrofolliculomas since age 40 years		

a Described by Leter *et al*, 2008.

b In BHD 2 an exon 1 deletion was detected in addition to two intronic *FLCN* variants, IVS9+6 C>T and IVS8+36G>A. Variant IVS9+6 C>T was previously reported in a patient with suspected BHD who had multiple renal tumours (Gatalica *et al*, 2009). Both variants however, have since been reported to be rare polymorphisms (https://grenada.lumc.nl/LOVD2/shared1/home.php?select_db=FLCN).
